# Impacts of glacial discharge on the primary production in a Greenlandic fjord

**DOI:** 10.1038/s41598-024-64529-z

**Published:** 2024-07-30

**Authors:** Yasuhiro Hoshiba, Yoshimasa Matsumura, Naoya Kanna, Yoshihiko Ohashi, Shin Sugiyama

**Affiliations:** 1 Japan Agency for Marine-Earth Science and Technology, Yokosuka, Japan; 2 Atmosphere and Ocean Research Institute, The University of Tokyo, Kashiwa, Japan; 3 National Institute of Polar Research, Tachikawa, Japan; 4 Institute of Low Temperature Science, Hokkaido University, Sapporo, Japan; 5 Arctic Research Center, Hokkaido University, Sapporo, Japan

**Keywords:** Ocean sciences, Environmental impact, Element cycles

## Abstract

Subglacial discharge from marine-terminating glaciers in Greenland injects large volumes of freshwater and suspended sediment into adjacent fjord environments. Although the discharge itself is nutrient poor, the formation of meltwater plumes can enhance marine biological production by stimulating upwelling of nutrient-rich fjord water. Despite the importance of meltwater discharge to marine ecosystems, little is known of the quantitative impact of discharge processes on phytoplankton growth, including the effects of local plumes, fjord-wide stirring and mixing, and suspended sediments on net primary production (NPP). Here, we report simulations of Bowdoin Fjord in northwestern Greenland using coupled non-hydrostatic ocean circulation and lower-trophic level ecosystem models, developed using field data. Our findings demonstrate that subglacial discharge plays a crucial role in NPP by stirring and mixing the entire fjord water system, which has a greater effect on NPP than local plume upwelling. Sensitivity tests suggest a 20% increase in NPP under conditions of enhanced discharge anticipated in the future. However, if glacier discharge and retreat exceed critical levels, NPP is predicted to decline by 88% relative to present values. This pattern reflects the negative impact of increased sediment flux on photosynthesis and weakened fjord stirring and mixing resulting from shallower outlet depths.

## Introduction

**Figure 1:**
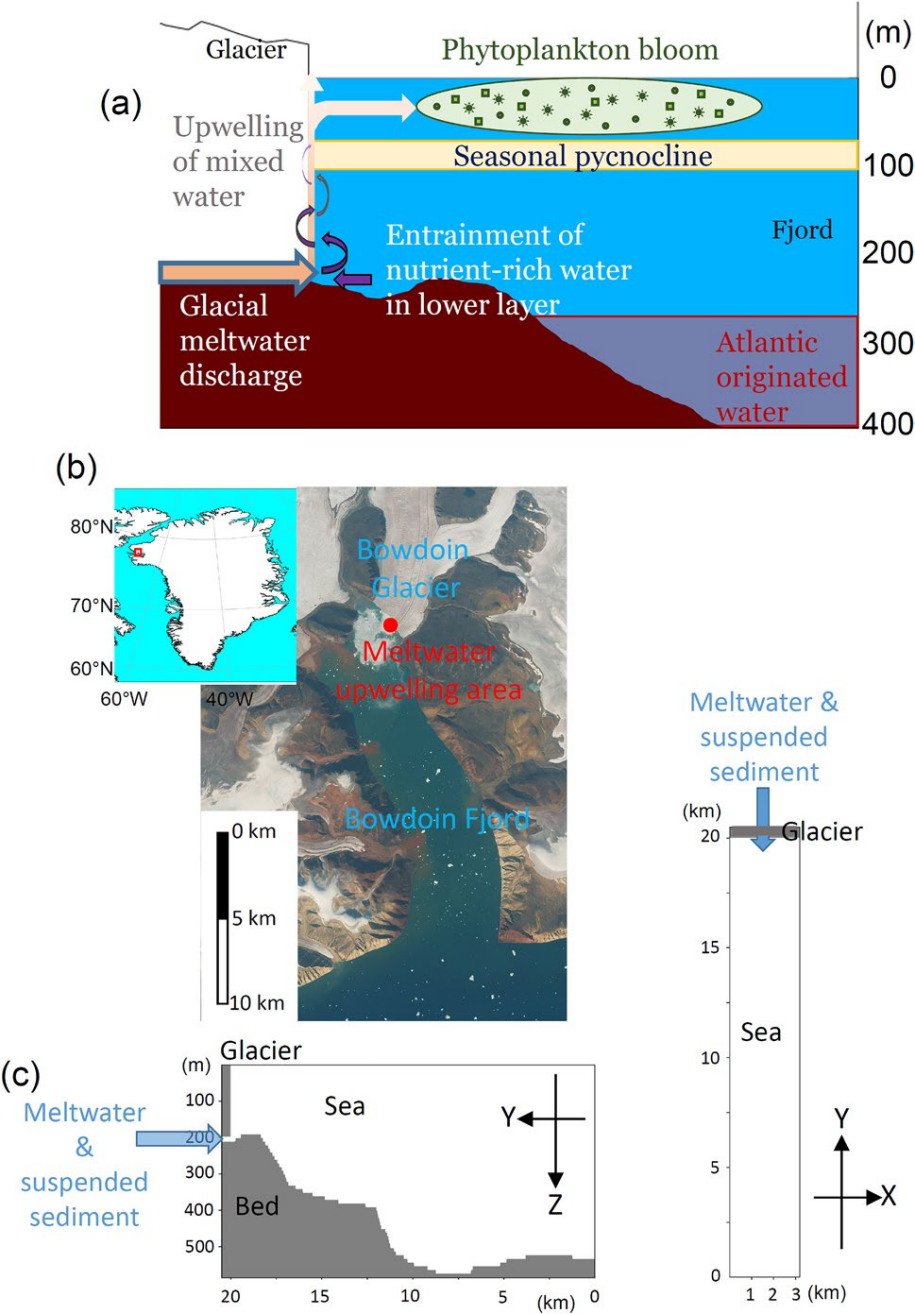
(**a**) Schematic showing how subglacial discharge influences the formation of nutrient-rich subsurface plume water and the occurrence of phytoplankton blooms in Bowdoin Fjord. (**b**) Bowdoin Glacier and its fjord in northwestern Greenland (red box, inset). (**c**) Vertical cross-section and plan view of the model topography, in which sediment-laden freshwater is discharged from a glacier terminus situated at the northern boundary of the model (blue arrow).

Among the > 1500 calving glaciers in the Northern Hemisphere, nearly 80% have retreated in recent years, losing a total area of 7527 ± 31 km^2^ between 2000 and 2020^1^. More than half of this mass loss has occurred at the Greenland Ice Sheet, where numerous outlet glaciers terminate in fjords^2^. The Greenland Ice Sheet lost 3,902 ± 342 Gt of ice between 1992 and 2018, and the rate of ice loss has increased over time^3^. Meltwater produced on the glacier surface is transported to the glacier bed and onward to the glacier front via a subglacial drainage system. At the termini of marine-terminating glaciers, large amounts of freshwater and suspended sediment are discharged directly from the glacier bed into the marine environment, whereupon the discharge entrains relatively warm, high-salinity deeper waters^4^ and upwells along the glacier front as a plume (Fig. 1a). In the case of glaciers terminating in deep fjords, the buoyancy of the plume declines as it rises, such that only a fraction of the upwelling water reaches the sea surface^5^ or equilibrium depth^2^. Most of the upwelling water spreads offshore through the sub-surface layer, supplying suspended sediment and nutrients to the upper layers of the fjord. This process can result in highly turbid water occupying the sub-surface layer^6,7^, while the deeper, nutrient-rich water entrained by glacial discharge^8,9^ enhances biological production in the surface layer during the summer melt season^10–12^. Via these processes, therefore, meltwater discharge from marine-terminating glaciers exerts a significant impact on biological production, hydrological cycling, and mass transport in Greenlandic fjords^13–15^.

Subglacial discharge can dramatically increase nitrate concentrations in fjord surface layers during the summer melt season. As nitrate is a limiting nutrient for phytoplankton in most sectors, upwelling nitrate-rich deep waters often result in summer phytoplankton blooms^16,17^. Conversely, the influx of highly turbid glacial discharge can also reduce the availability of light, thereby limiting phytoplankton growth^18^. Although the importance of glacial meltwater discharge in fjord environments has been established, quantitative assessments of its impact on marine biogeochemistry remain sparse. For instance, Slater et al.^2^ applied analytical modeling to evaluate the fluxes of upwelling plumes water under different magnitudes of meltwater discharge and depths of the subglacial outlet; Hopwood et al.^19^ used a theoretical model to estimate the biological influence of nitrate fluxes from upwelling plumes. Oliver et al.^20^ discussed nutrient export out of the fjord by simulation using a parameterization and approximation for plumes. To date, however, few studies have sought to numerically model the impact of meltwater-driven nutrient supply on primary production in the surficial fjord environment. This gap in our knowledge potentially reflects the general dearth of oceanographic data from near glacier fronts, which hinders the evaluation of model performance. A notable exception is Bowdoin Fjord in northwestern Greenland, for which observational data (suspended sediment concentration and particle size, chlorophyll, and nutrient content) have been obtained immediately adjacent to the calving terminus of Bowdoin Glacier^16^.

In this study, we employ a lower-trophic level ecosystem model (Supplementary Fig. 1a) coupled with a non-hydrostatic ocean circulation model to simulate summertime biological production in Bowdoin Fjord (Fig. 1b). In other current simulation studies of meltwater plumes from subglacial discharge, most use hydrostatic models and parameterizations. Our non-hydrostatic model can effectively reproduce vertical motions within the water column, including meltwater plumes from subglacial buoyancy inputs. Our ecosystem model includes suspended sediments that may inhibit photosynthesis, and we apply a novel technique of dividing the nutrient flux into four sources (Supplementary Fig. 1b). The coupled ocean model enables quantification of the processes linking the meltwater flux from Bowdoin Glacier to the physical field, mass transport, and net primary production (NPP) in Bowdoin Fjord. To the authors’ knowledge, no study has quantitatively and comprehensively analyzed the characteristic physical processes (i.e., plume upwelling, stirring, and mixing) with respect to phytoplankton proliferation in a fjord, including the processes of nutrient supply for NPP initiated by meltwater discharge. We also conduct sensitivity experiments (see Table 1 and “Methods”) to estimate the effects on NPP of variability in meltwater discharge and glacier recession due to climate change. In terms of physical and glaciological settings and nutrient (nitrate) concentrations^21^, Bowdoin Fjord is similar to other Greenlandic glacier fjords hosting marine-terminating systems. Therefore, our results contribute to the general understanding of NPP in glacial fjords throughout Greenland.

**Table 1:** Glacial discharge parameters employed in the sensitivity experiments.

Meltwater discharge (m^3^/s)	0 (WOD)	12.5	25	50 (STD)	100	200
Depth of outlet (m)	0	50	100	150	200 (STD)	

STD denotes the combined parameters of 50 m^3^/s discharge and 200 m outlet depth estimated from previous studies^2,9,22,43^. WOD denotes the case without meltwater discharge.

## Results

### Effect of glacial discharge on primary production

**Figure 2:**
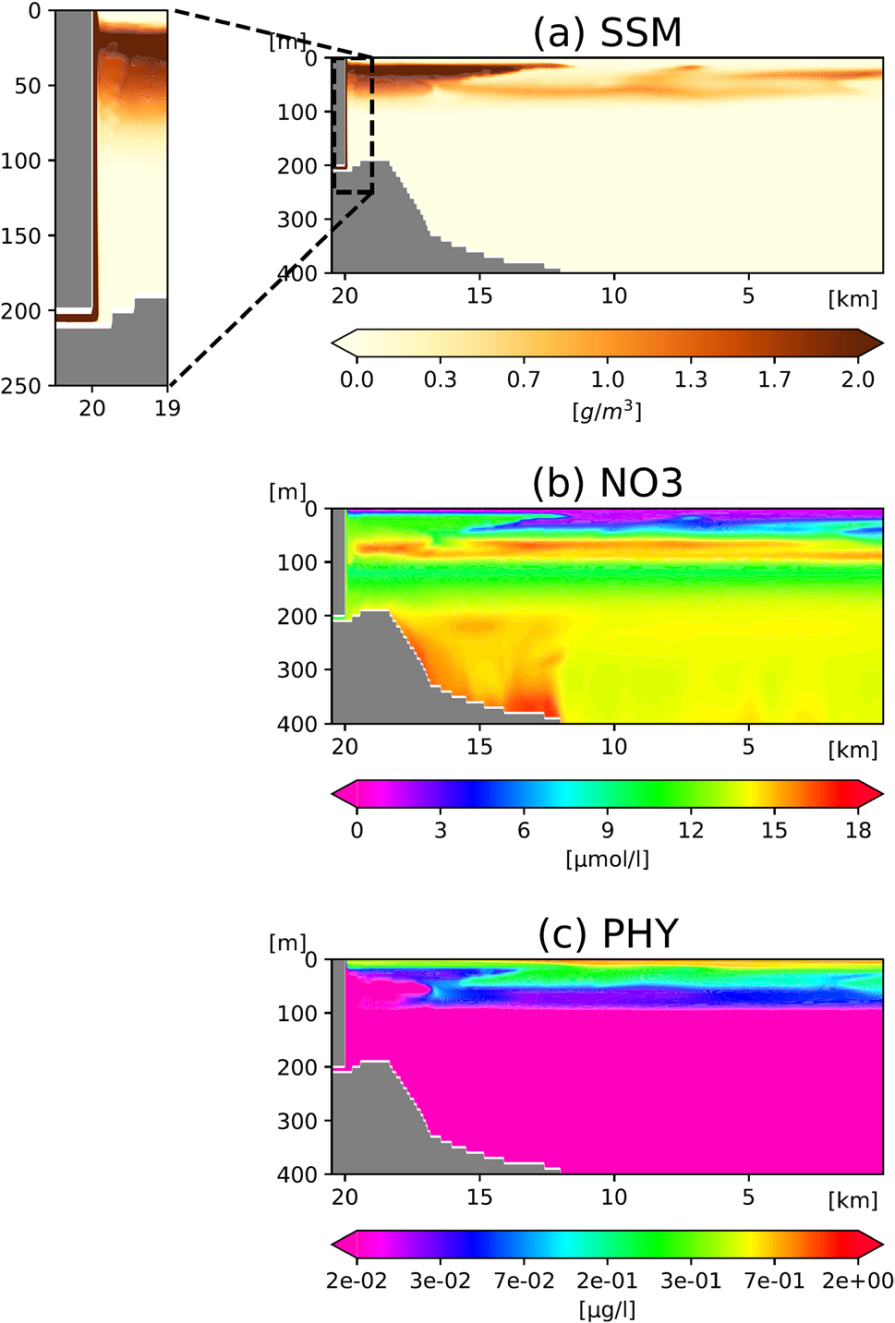
Vertical cross-sections of the model domain, depicting the distributions of STD (**a**) suspended sediment matter (SSM), (**b**) nitrate (NO3), and (**c**) phytoplankton (PHY) after 19 days since the onset of the meltwater discharge. The ecosystem model runs on a nitrogen basis. Unit conversion from nitrogen (μmol/l) to chl.*a* (μg/l) follows Ref.^44^.

We evaluate the standard case (STD; see Table 1 and “Methods”), in which meltwater discharge (50 m^3^/s) and outlet depth (200 m) are derived from the modern estimations and observations of mid-summer discharge conditions at Bowdoin Glacier^2,22^. Suspended sediment is injected into the fjord at a depth of 200 m, whereafter it upwells along the length of the calving front. As described above, however, only a fraction (< 1%) of that sediment reaches the sea surface; the majority (> 99%) is distributed within the upper layer (1–100 m depth) (Fig. 2a). Of the water arriving at the surface, only 2%–3% is derived from glacial discharge, with the remainder comprising ambient seawater that has been entrained *en route*. These results are consistent with prior observations of Greenlandic fjords, which reported glacial and entrained components of surficial plume water of 7%–10% and 90%, respectively^8,9^. Field studies in Bowdoin Fjord have also identified stratification in the upper 100 m during the summer melt season^22^. Prior to meltwater input, nitrate concentrations in this upper layer are low (Supplementary Fig. 2a). Subsequent entrainment of nitrate-rich fjord water by glacial discharge causes nutrients to be supplied to the upper layer via the upwelling plume. (Fig. 2b). However, although phytoplankton growth can be stimulated by an enhanced nitrate flux (Fig. 2c), the high concentration of suspended sediment (> 1 g/m^3^) in the glacial plume reduces light availability in the upper water, thereby limiting the viability of phytoplankton in the nutrient-rich waters within 15–20 km of the glacier terminus.

**Figure 3:**
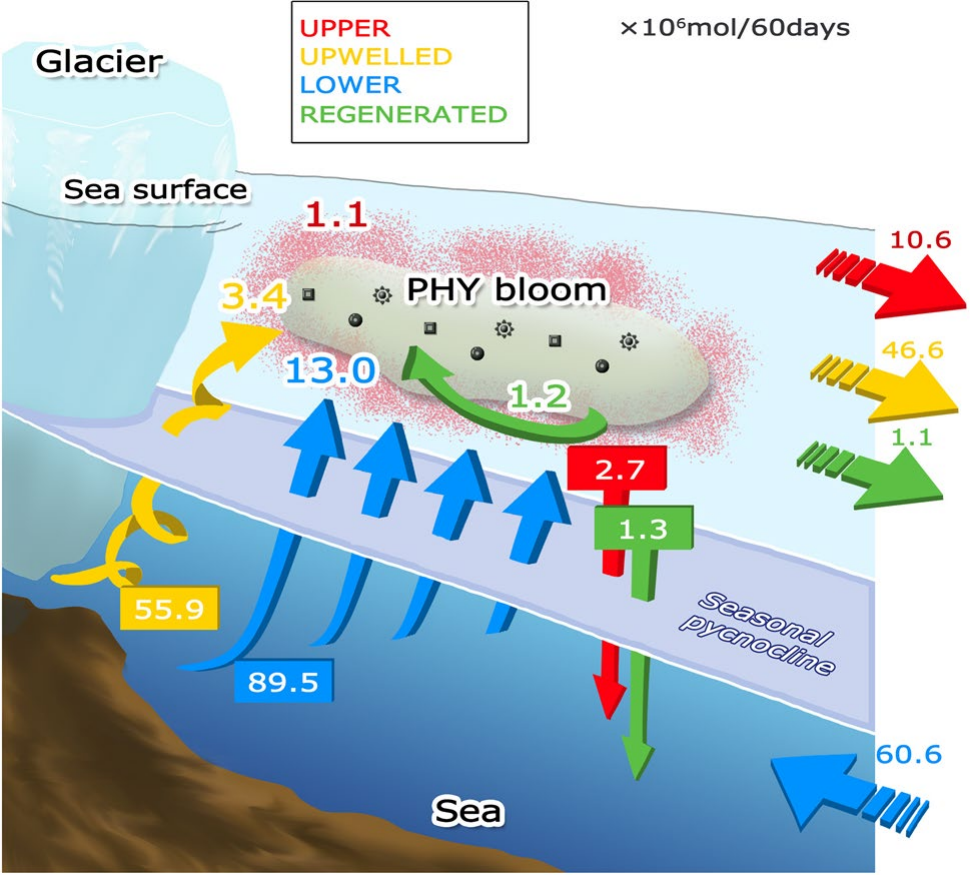
Schematic representation of the nitrate budget associated with subglacial discharge and phytoplankton blooms. Numbers outlined in white around “PHY bloom” indicate the net primary productions of each nitrate source (here synonymous with nitrates used for phytoplankton). Numbers enclosed in squares indicate the amounts of nitrates transported across the seasonal pycnocline at approximately 100 m depth. Numbers to the right indicate the amounts of nitrates leaving or entering the fjord. All the numbers represent the difference between STD and WOD for 60-day model runs (× 106 mol/60 days).

In addition to meltwater plumes, other processes might also contribute to the vertical transport of nitrate within the fjord water column. For instance, nitrate is known to be supplied by vertical motion associated with density-driven circulation^23^, which arises from the density gradient between the low-salinity (low density) calving zone to the high-salinity (high-density) offshore marine zone. Upwelling plumes are also believed to weaken stratification of the entire fjord system, thereby enhancing turbulent vertical mixing. We conducted a series of experiments to assess the relative importance of these various nitrate-transport processes for fjord productivity. To examine the sources of nitrate exploited by phytoplankton blooms, we divided the total amount of nitrate into four components (Supplementary Fig. 1a, b): UPPER nitrate, initially present above 100 m depth; UPWELLED nitrate, initially present below 100 m depth but transported above 100 m by the upwelling meltwater plume; LOWER nitrate, comprising the remainder of the nitrate initially present below 100 m; and REGENERATED nitrate, which has been utilized by the ecosystem more than once and re-mineralized. Both UPWELLED and LOWER nitrates are nutrients that initially reside in the lower layer. However, the intention is to distinguish between nutrients that rise directly near the glacier as a result of the subglacial discharge plume and those that are derived from the lower layer by indirect flows distant from the glacier (for details, see “Nutrient source separation” in the “Methods” section). During the discharge period, the four nitrate components exhibit different distributions within the fjord (Supplementary Fig. 3) and thus play roles of differing importance in surficial phytoplankton growth. We compute the contribution of each nitrate source to NPP; the budget over a 60-day period is summarized in Fig. 3.

As our objective is to quantify the impact of glacial discharge on the primary production in Bowdoin Fjord, we describe the contributions of the four nitrate components to NPP in the STD as their respective differences from a case without discharge (WOD in Table 1). These experiments indicate that the total NPP difference is 1.1 for UPPER nitrate, 3.4 for UPWELLED nitrate, 13.0 for LOWER nitrate, and 1.2 for REGENERATED nitrate (units in × 10^6^ mol). UPWELLED and LOWER nitrates account for 88% of the total uptake for phytoplankton growth. During the experiment, 55.9 × 10^6^ and 89.5 × 10^6^ mol of nitrates were transported to the upper layer as UPWELLED and LOWER nitrates, respectively, whereas 2.7 × 10^6^ of UPPER nitrate and 1.3 × 10^6^ mol of REGENERATED nitrate were exported to the lower layer. The total amount of nitrate supplied to the upper 100 m by subglacial discharge, directly and indirectly, is 145 × 10^6^ mol. These results are critical to understanding the nutrient supply in the fjord because, in addition to the direct upwelling of nitrate via the meltwater plume, nitrate supply by the density-gradient-driven circulation and enhanced vertical turbulent mixing over the entire fjord are essential for the phytoplankton bloom during the summer melt season. The density-gradient-driven circulation forces flow offshore within the surface layer and glacier-ward flow in the lower layer, thereby drawing LOWER nitrate into the fjord system (within the computational domain) and exporting it to the upper layer. Concurrently, UPPER, UPWELLED, and REGENERATED nitrates above 100 m depth are exported from the fjord. The total net inflow of nitrate is + 2.3 × 10^6^ mol, indicating that the total amount of nitrogen in the fjord, including the ecosystem itself, increases relative to the WOD owing to the combined effects of fjord productivity and enhanced circulation, both of which are linked to glacial meltwater discharge.

### Impacts of increasing discharge and glacier retreat on primary production

**Figure 4:**
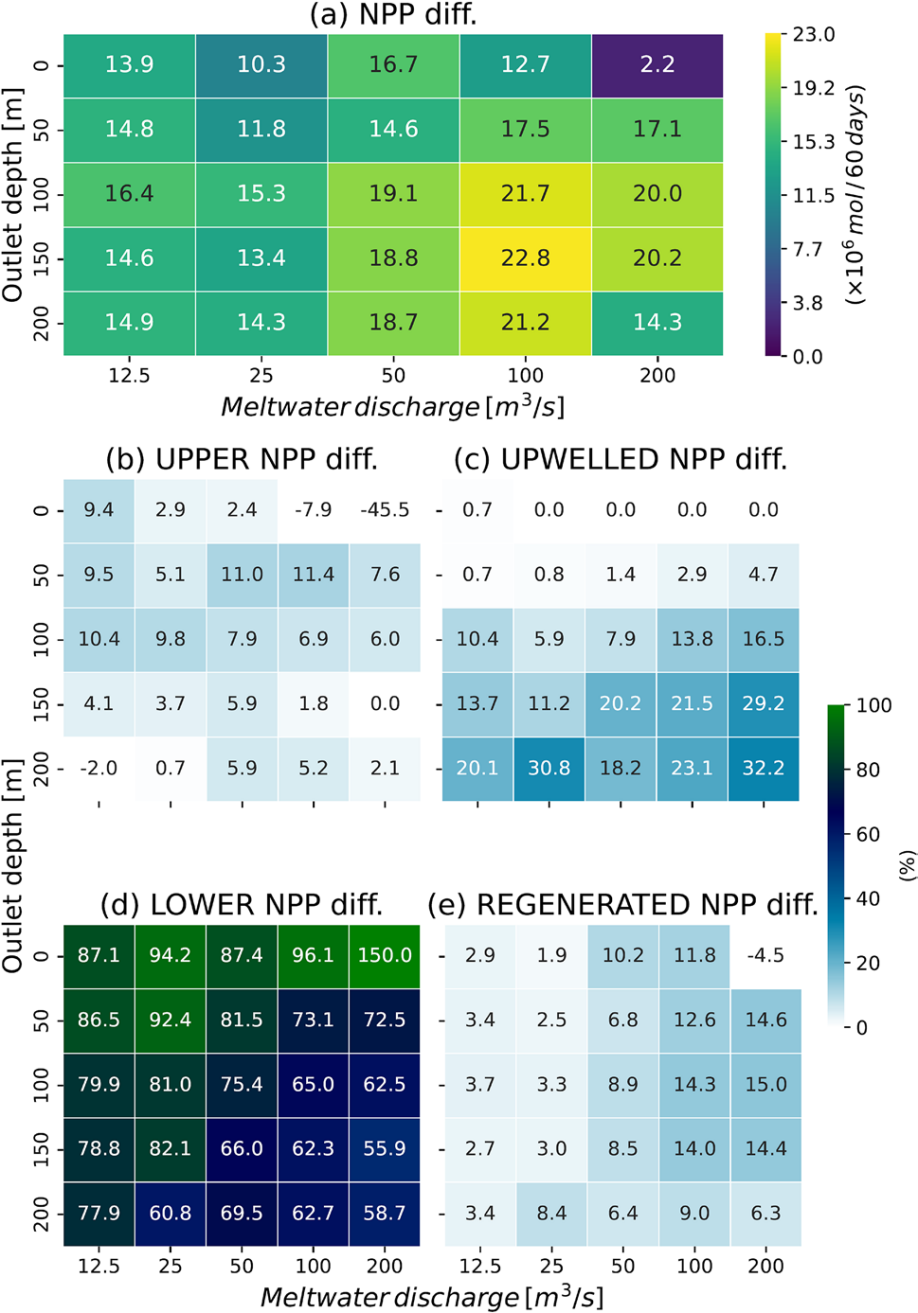
(**a**) Sensitivity experiment results with respect to changes in discharge (x-axis) and outlet depth (y-axis). The presented NPPs are valued relative to those obtained in WOD, integrated over the entire model domain, and accumulated for the 60-day model runs. Panels (**b**,**c**,**d**,**e**) are the same as in (**a**) but for UPPER, UPWELLED, LOWER, and REGENERATED contributions, respectively.

The foregoing analysis demonstrated the impact on fjord NPP of glacial discharge under current conditions (STD: discharge 50 m^3^/s, outlet depth 200 m). But what will happen if the rate of glacial melt increases and the calving front retreats owing to climate warming? To address this question, we conducted sensitivity experiments (Table 1) incorporating different meltwater discharge and subglacial outlet depths (Fig. 4). As discharge increases to 100 m^3^/s, but with the outlet depth set at 150 m, we observe NPP increasing by a factor of 1.2 (Fig. 4a). However, as discharge increases further and the glacier margin retreats out of the marine environment (discharge 200 m^3^/s, outlet depth 0 m), NPP declines markedly to values as low as 1/8–1/9 of the modern value.

Multiple factors can be invoked to explain this pattern. With a few exceptions (see below), the NPP of phytoplankton blooms increases with rising discharge and deepening outlets. Although the dominant nutrient source for phytoplankton is LOWER nitrate (Fig. 4d), the relative importance of UPWELLED nitrate (Fig. 4c) grows with increasing discharge and outlet depth. In a few cases, however, NPP fractions associated with UPPER (Fig. 4b) and REGENERATED (Fig. 4e) nitrates exhibit negative values, demonstrating a negative impact of meltwater discharge on primary production. In such cases, the outlet is located at the surface, implying that nitrates in the upper layer are exiting the fjord before being fully consumed by phytoplankton.

**Figure 5:**
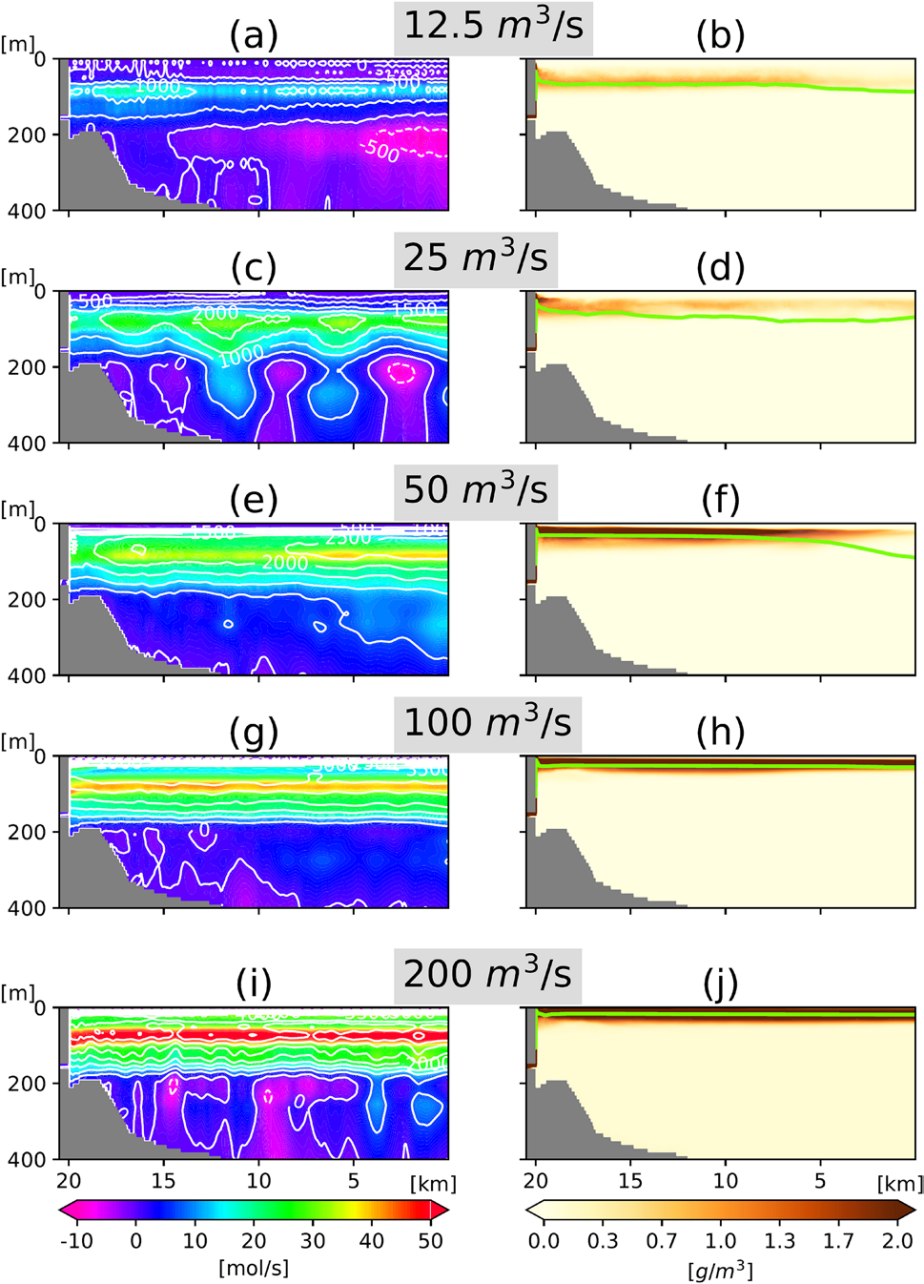
Stream function (contour line) and total nitrate flux (color contour) along the north–south section obtained 15–19 days (mean) after the onset of discharge from a 150 m deep outlet of (**a**) 12.5, (**c**) 25, (**e**) 50, (**g**) 100, and (**i**) 200 m^3^/s. Contour intervals for stream function are 500 m^3^/s. Solid and dotted contour lines represent positive and negative numbers corresponding to clockwise and counterclockwise circulations, respectively. Positive (negative) values of nitrate flux represent the amount of nitrate circulating clockwise (counterclockwise). Panels (**b**,**d**,**f**,**h**,**j**) show suspended sediment concentrations; light green lines indicate the depths at which the light intensities are 1% those at the surface.

In Fig. 5, the contours in the lefthand panels depict the strength of vertical circulation: positive values (solid contours) denote a predominantly clockwise vertical circulation that becomes amplified as discharge increases. Fjord circulation is enhanced by discharge because, similar to the estuarine circulation, the vertical motion is driven by low-density water on the glacier side and high-density water offshore^24^. Nitrate fluxes (colored contours) transported by this circulation also increase with discharge, indicating a clear increase in the supply of nitrates from lower to surface layers. However, rising nitrate fluxes do not necessarily result in elevated NPP. The righthand panels in Fig. 5 show concentrations of suspended sediment, which are seen to intensify near the surface as meltwater discharge increases. High concentrations of suspended sediment block the vertical penetration of sunlight such that penetration depth (light-green line in Fig. 5, righthand panels) decreases with increasing discharge. Since photosynthesis is limited by the availability of light, productivity can decline even under optimal nitrate levels. Consequently, when outlet depth is held steady (Fig. 4a), NPP peaks at 50–100 m^3^/s and declines under the maximum discharge conditions (200 m^3^/s).

When discharge is held steady but outlet depth decreases, NPP exhibits a progressive decline owing to the shallowing of vertical circulation and reduction in nitrate transport (Supplementary Fig. 4). The shallower the outlet, the smaller the nitrate flux to the surface and the lower the NPP (Fig. 4a). This result is consistent with previous research showing how fjords with marine-terminating glaciers are more productive than those with land-terminating glaciers^17^. Nevertheless, our results confirm earlier findings (Ref. 19) that NPP drops when the outlet depth is too deep. In our sensitivity experiments with fixed discharge amount and variable outlet depth (Fig. 4a), the highest NPP coincides with outlet depths of 100 and 150 m; NPP is lower for the deeper outlet (200 m). Prior work attributed this outcome to the reduced likelihood of plume water reaching surface layers when injected at great depth^19^. However, our results indicate that enhanced vertical circulation is responsible for the reduction in NPP, such that greater amounts of phytoplankton are transported to lower layers where light levels are insufficient for photosynthesis. We note that the fraction of NPP due to UPWELLED nitrate (Fig. 4c) is highest when the outlet depth is 200 m, suggesting a significant portion of plume water reaches the surface layers. Therefore, although this study and ref.^19^ share the same conclusion about the impact of discharge depth on NPP, our results highlight the importance of incorporating biological processes into simulations of the role of glacier discharge in primary production.

## Discussion and remarks

We first estimated the impact of the current subglacial meltwater discharge on the ecosystem of Bowdoin Fjord. An analysis of the separate sources of nutrient uptake by phytoplankton demonstrated that the effect of the indirect transport of nutrients by freshwater buoyancy stirring the entire fjord is more important to phytoplankton growth than the direct export of nutrients by the glacial meltwater plume to the upper layers. Under the current discharge conditions, the entire water body of the fjord is involved in circulation, and nutrients drawn in from the lower part of the area outside of the fjord result in a positive nutrient budget during the summer melt season that would sustain the ecosystem in other seasons. This pattern implies that if the direct effect of upwelling plumes were the only control on flows in the fjord, then the nutrients in the fjord would be depleted over the long term.

We then conducted sensitivity experiments with different discharge rates and outlet depths. The results show that when discharge increases and the outlet becomes shallower (glacier retreat) owing to climate change, the NPP in the fjord temporarily increases to 1.2 times the current level, but when the change is extreme, the NPP decreases to 0.1–0.2 times the current level. In the case of the most extreme suppression of phytoplankton growth, the outlet depth is 0 m and the discharge is 200 m^3^/s. Under such conditions, the glacier is no longer a marine-terminating glacier but rather a land-terminating glacier that feeds a river. As the outlet depth shallows, nutrient drawdown from outside the fjord also tends to weaken. This implies a long-term depletion of nutrients in the fjord.

Regarding the robustness of the simulation, the physical part of the model is based on previously published settings^22^, and we confirmed that the model output is in reasonable agreement with reality, especially in terms of the vertical distributions of temperature and salinity. The results of the ecosystem component of the simulation were compared with limited observation data (see “Methods” section), and we consider that the observed vertical profiles are reasonably reproduced. However, given the discrepancies between ecosystem simulations and observations, we also conducted experiments with different initial NPZD concentrations, although the full results are not presented here. For example, in an experiment in which the initial NPZD level of the entire ecosystem was increased by a factor of 1.5, the four nitrates required for the NPP of interest in this study did not rank differently from those in the STD, and the effect of each nitrate on NPP was within 2% of that in the STD. Therefore, the main conclusion of this study would not change even if the distribution of the ecosystem component were to change slightly.

The discharge rate and outlet depth have large uncertainties, which were addressed by conducting sensitivity experiments in which these properties were varied within a realistic range. Modeling that considers variations in the lateral (e.g., azimuthal) direction of the outlet (x-direction in Fig. 1c) and the number of outlets is beyond the scope of this study, but these possible variations would not substantially affect the present conclusion that the buoyant inputs of meltwater stirring the entire fjord has a greater effect on NPP than local plumes. This is because the vertical circulation is driven by the density difference between waters on the glacier side of the fjord and those on the offshore side, and the strength (stream function) is calculated by integrating the water velocities in the fjord. Thus, varying the x-direction discharge conditions might slightly change the NPP of UPWELLED nitrate contributed by local plumes, but would not be expected to significantly change the NPP of the critical LOWER nitrate.

We also suspect that under extreme conditions, NPP is affected by suspended sediment transported via the upwelling plume. To assess the scale of this effect, we performed additional experiments without suspended sediment (Supplementary Fig. 5). Simulations in which outlet depth is 0 m showed consistently greater NPP than those incorporating suspended sediment (Fig. 4a), thereby implying a significant impact of suspended sediment on light availability in the water column. The simulations used in this study assume a constant sediment concentration in the meltwater. However, no simple relationship has been observed between meltwater discharge and sediment concentrations for glaciers in other Greenlandic fjords^25^. If the sediment concentration of glacial discharge increased with rising meltwater rate, then the decline in NPP due to light attenuation might be even more severe. The relationship between suspended sediment concentration and meltwater discharge will be better constrained once further observations are available.

Recognizing that our experiments were parameterized by data specific to Bowdoin Fjord, a number of sensitivity experiments indicate that our conclusions are applicable to other glaciated fjords both in Greenland and beyond. However, we note that the summer Bowdoin Fjord (the focus of this study) is a fjord with the following characteristics. First, it is deeper than the typical continental shelf depth of 200 m (Fig. 1a), which makes it more likely to draw in warm, salty water of North Atlantic origin at lower levels. Second, the depth at which discharged water acquires neutral buoyancy during upwelling (neutral buoyancy depth) tends to be shallower than the euphotic depth (Fig. 5). According to a previous study^2^, ~ 60% of the representative tidewater fjords in Greenland have an effective depth of > 200 m, as in this study, and that ~ 87% of the fjords have a neutral buoyancy depth that may reach the upper euphotic layer. The above depths are strongly related to glacial meltwater discharge and phytoplankton proliferation^26^, and the characteristics of Bowdoin Fjord are shared by many Greenlandic fjords. However, Bowdoin Fjord has fewer icebergs than other fjords of similar size, which enables boats to approach the glacier front. The physical and biogeochemical conditions near the surface may be different in fjords where the sea surface is populated by icebergs. Bowdoin Fjord is part of the larger outer part of the Inglefield Bredning Fjord system, which we are currently observing and simulating. We anticipate future work on other cases, such as large fjords, shallow fjords, and fjords where the neutral buoyancy depth does not reach the euphotic layer.

Greenlandic glaciers are undergoing intense melting events owing to atmospheric warming in Arctic latitudes, as documented by the long-term trend of rising annual meltwater flux^27^. Furthermore, glaciers in northwestern Greenland, including Bowdoin Glacier, have lost considerable mass since 2000^28^, with further change expected in the coming decades. Changes in the biological production of fjords due to the retreat of marine-terminating glaciers are a concern not only in Greenland^17,19^ but also in Svalbard^29^, the Canadian Arctic Archipelago^30,31^, and the Antarctic Peninsula^32,33^. Anthropogenic climate change is expected to have pronounced impacts on the high latitudes. Considering the widespread shrinkage of marine-terminating glaciers, any associated change in NPP will undoubtedly play an important role in polar marine ecosystems.

## Methods

### Model description

Bowdoin Glacier (77.6°N, 66.8°W) is a marine-terminating outlet glacier in northwestern Greenland that discharges freshwater and suspended sediment into a fjord (Bowdoin Fjord) that is approximately 3 km wide and 20 km long (Fig. 1). Water depth is approximately 600 m at the fjord entrance and shallows to 210 m at a distance of one kilometer from the glacier terminus. Based on the observations near the glacier front in 2013, the glacier is grounded, and between 86 and 89% of the ice lies below sea level^34^. Sea ice disappears from the fjord in early July and the summer melt season is characterized by upwelling meltwater plumes that are observable as highly turbid zones along the glacier front^16^.

Meltwater discharge into Bowdoin Fjord is simulated by a non-hydrostatic ocean model^35^. The detailed physical setup follows the 2016 case of Ohashi et al.^22^ (hereafter OH20) and OH20 confirms that the physical fields (e.g., vertical profiles of temperature and salinity) of Bowdoin Fjord during the summer are effectively reproduced. OH20 assumes initially quiescent conditions of horizontally homogeneous stratification based on the temperature and salinity estimated from observations, followed by the inflow of meltwater into the fjord. Tidal effects from outside the fjord are not considered. The differences between OH20 and this study are as follows: the vertical resolution of the upper layer is increased from 5 to 1 m for depths < 10 m, and to 2 m for depths between 10 and 20 m; the associated changes in initial water temperature and salinity; the presence of restoration to external physical conditions at the fjord mouth; and the amount of meltwater discharge and the calculation period. In OH20, water temperature and salinity were restored at the fjord entrance, whereas no restoration is applied for this study so as to estimate more accurately the budget of material entering and leaving the fjord. The meltwater discharge varies within a plausible range (see “Sensitivity experiments and analysis”), whereas the calculation period is extended from 7 days in OH20 to 60 days (19 days of meltwater discharge + 41 days without discharge). The rationale for extending the calculation period is that phytoplankton growth takes time and this study focuses specifically on NPP. Moreover, it takes up to two months for the summer ocean-stratification conditions around Greenland to change^36,37^, and our experiments assumed a 60-day summer melting period. The duration of the meltwater discharge (19 days) was determined from previous studies^9,22^. Here, we focus specifically on the flooding period. The summer period with daily discharge exceeding the July 2016 average was 19 days at Bowdoin Glacier. As preliminary experiments, several simulations were performed that varied the discharge duration but not the total discharge (not shown here), on which basis the main conclusions remained the same, and the 19 day discharge period was adopted because it gave the most reasonable results. As a result of the above changes, we consider simulations to produce a more realistic reproduction of conditions within Bowdoin Fjord.

This study employs an NPZD-type lower-trophic level ecosystem model^38^, which is parameter-tuned to a Northern Hemisphere, high-latitude marginal sea and seasonal-sea ice zone. The ecosystem model runs on a nitrogen basis, which we consider reasonable because nitrate, as a source of nitrogen, is the primary limiting factor for summer phytoplankton growth in Bowdoin Fjord. The initial conditions of the ecosystem component (Supplementary Fig. 2a,b) were created as follows: the ecosystem part runs for five years in the initial physical field, with no discharge and flow, and the nitrate, plankton, and detritus distributions are set to a quasi-steady state. Nitrate is lowest in the surface layer and increases with depth, and phytoplankton are present at depths of < 100 m where sunlight is available. Solar irradiation is prescribed as 276 W/m^2^ at the sea surface, corresponding to the surface downward solar radiation in July at the latitude of Bowdoin Fjord^39^. The light parameter is held constant for the 60-day-long simulation, assuming white nights. For a case without discharge (WOD in Table 1), the initial conditions described above persisted for 60 days. As NPP by phytoplankton occurs even in the absence of subglacial meltwater discharge, the differences from WOD are discussed to estimate the impact of discharge on NPP. Although it is not possible in reality to have no flow, the decision to make the initial conditions and WOD a static state enabled long-term simulations, comparisons, and differentiation. Based on the available computational resources and to ensure comparability, we adopted the static situation.

Following these initial distributions, discharge of glacial meltwater is introduced into the simulations. Discharge rates and depths vary, as outlined above. The suspended sediment concentration in the meltwater is fixed at 132 g/m^3^. Sediment settling occurs at a rate of 1.27 × 10^−5^ m/s, and suspended sediment is removed from the fjord water at a rate of 1.5 × 10^7^ s (i.e., the concentration of suspended sediment is reduced to 1/e within ~ 170 days). These values are estimated from the concentration and particle size of suspended sediment in the plume water collected at Bowdoin Fjord in 2016^16^. Phytoplankton photosynthesis is inhibited by suspended sediment concentrations; the intensity coefficient for shading follows ref.^40^ for turbid river water.

Our simulation results are compared with observations at Bowdoin Fjord in July 2016. The observation period was several weeks after peak discharge, according to glacier measurement and meteorological conditions. For full details of the observational data, we refer readers to a previous paper^16^. The vertical profiles of nitrate and chlorophyll concentrations used in the STD simulations agree well with the observational data (Supplementary Figs. 6 and 7). For chlorophyll concentrations, model simulations do not reproduce the absolute values of the concentration maxima at the very surface reported by field observations, although vertical distributions are captured with sufficient accuracy to draw our conclusions.

### Nutrient source separation

To quantify nitrate budgets during phytoplankton blooms, we delimit four nitrate sources according to their initial locations (Supplementary Fig. 1a,b). The first is nitrate derived initially at depths of < 100 m (UPPER nitrate), and the second is nitrate derived from > 100 m but supplied to depths < 100 m by the upwelling meltwater plume (UPWELLED nitrate). The third component (LOWER nitrate) incorporates the remainder of that initially present below 100 m depth; the portion of LOWER nitrate transported to the upper layer within 500 m of the glacier terminus is referred to as UPWELLED nitrate. The distance of 500 m was determined from the horizontal extent of plumes reproduced by the simulations (surface salinity > 29.6, suspended sediment concentration > 0.8 g/m^3^). Actual subglacial meltwater contains very little nitrate. To distinguish between the direct transport of nitrate (UPWELLED nitrate) in the meltwater plume and the indirect transport of nitrate (LOWER nitrate) by vertical circulation throughout the entire fjord, we divided the nitrate supplied from the lower layer (depth > 100 m) into two components: near the glacier (< 500 m from the glacier) and distant from the glacier (> 500 m from the glacier). The thickness of the upper layer characterized by phytoplankton blooms (100 m) is based on the depth of a pycnocline and the observed transition of water column properties in Bowdoin Fjord^22^. The fourth component (REGENERATED nitrate) is nitrate utilized by the ecosystem and then re-mineralized. Consequently, only the UPPER and LOWER components are present prior to glacial meltwater being introduced to the model. Thereafter, the amounts of UPWELLED and REGENERATED nitrate increase. The method of separating nutrient components has been used in previous studies of flooded rivers^41,42^, but a method such as that used in the present study, in which LOWER nitrate is converted to UPWELLED nitrate periodically in one area, is novel and suitable when considering episodic glacial meltwater discharge, which contains few nutrients.

### Sensitivity experiments and analysis

We conducted sensitivity experiments (Table 1) by changing the glacier meltwater discharge and outlet depth in order to investigate their effects on NPP. For the STD, discharge is 50 m^3^/s and the outlet depth is 200 m. In the sensitivity experiments, the discharge varies from 0 to 200 m^3^/s, and the outlet depth ranges from 200 to 0 m at 50 m intervals (total of 30 cases). The ranges for discharge and outlet depths used here were taken from previous studies of Greenland glaciers^2,9,22,43^, and the range of outlet depth (0–200 m) reflects the anticipated changes owing to ongoing glacier retreat. We did not consider changes in bathymetry because of the horizontal migration of the glacier front. In addition to the standard experiments using a constant concentration of suspended sediment, we also ran several experiments without sediment to investigate the influence of light shading on phytoplankton (Supplementary Fig. 5).

During meltwater discharge, suspended sediment, nitrate, and phytoplankton distributions differ in the eastern and western sectors of the fjord, partly reflecting the influence of Earth’s rotation. In this study, we focus on the vertical (y–z direction in Fig. 1c) distribution and do not discuss any variability in the east–west distribution (x–z direction in Fig. 1c). Nonetheless, we can confirm that our conclusions are not sensitive to east–west averaging since the budget analysis and stream function are integrated over the entire computational domain. The detailed cross-fjord (x–z) distributions are beyond the scope of this study.

### Supplementary Information

Supplementary Figures.

## Data Availability

Sunlight radiation data were obtained from the Numerical Prediction Division, Information Infrastructure Department; Japan Meteorological Agency, Japan (https://jra.kishou.go.jp/JRA-55/atlas/en/surface_ex.html). In situ nitrate and phytoplankton data can be downloaded from https://ads.nipr.ac.jp/dataset/A20170420-002. Owing to their large size (approximately 200 TB), the files necessary to reproduce our simulations (both initial and boundary files) and model output will be made available by the corresponding author upon request. The model used here (kinaco) is available online at http://lmr.aori.u-tokyo.ac.jp/feog/ymatsu/kinaco.git/.
