# Supplementary material for: Impacts of glacial discharge on the primary production in a Greenlandic fjord
Source: Sci Rep. 2024 Jul 30;14:15530. doi: 10.1038/s41598-024-64529-z (PMC11289466; doi:10.1038/s41598-024-64529-z)
Supplement: Supplementary file 1 — Supplementary Figures. [file 41598_2024_64529_MOESM1_ESM.pdf]

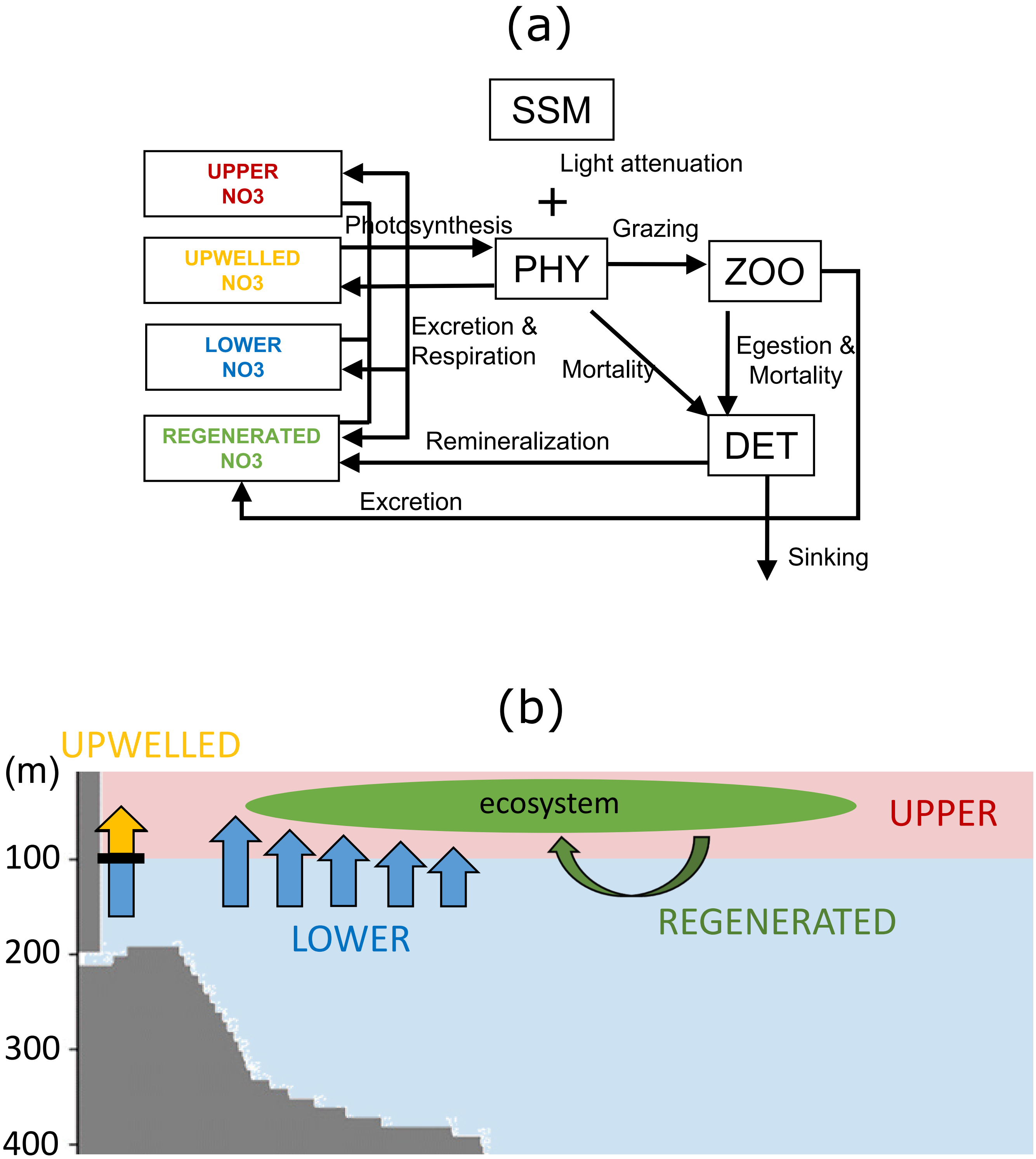


**Supplementary Figure 1.** (**a**) Schematic view of the nutrient source-separated ecosystem model. Nitrate utilized by phytoplankton blooms is divided into four categories: nitrate at depths < 100 m in the initial state (UPPER NO3); nitrate at depths > 100 m in the initial state, but transferred above 100 m by the upwelling meltwater plume (UPWELLED NO3); and the remainder of the nitrate initially present below 100 m (LOWER NO3). The fourth component is REGENERATED NO3, which is nitrate that has been used by the ecosystem and subsequently re-mineralized. (**b**) Schematic of the nutrient source separation. The portion of LOWER nitrate transported to the upper layer within 500 m of the glacier terminus is referred to as UPWELLED nitrate.

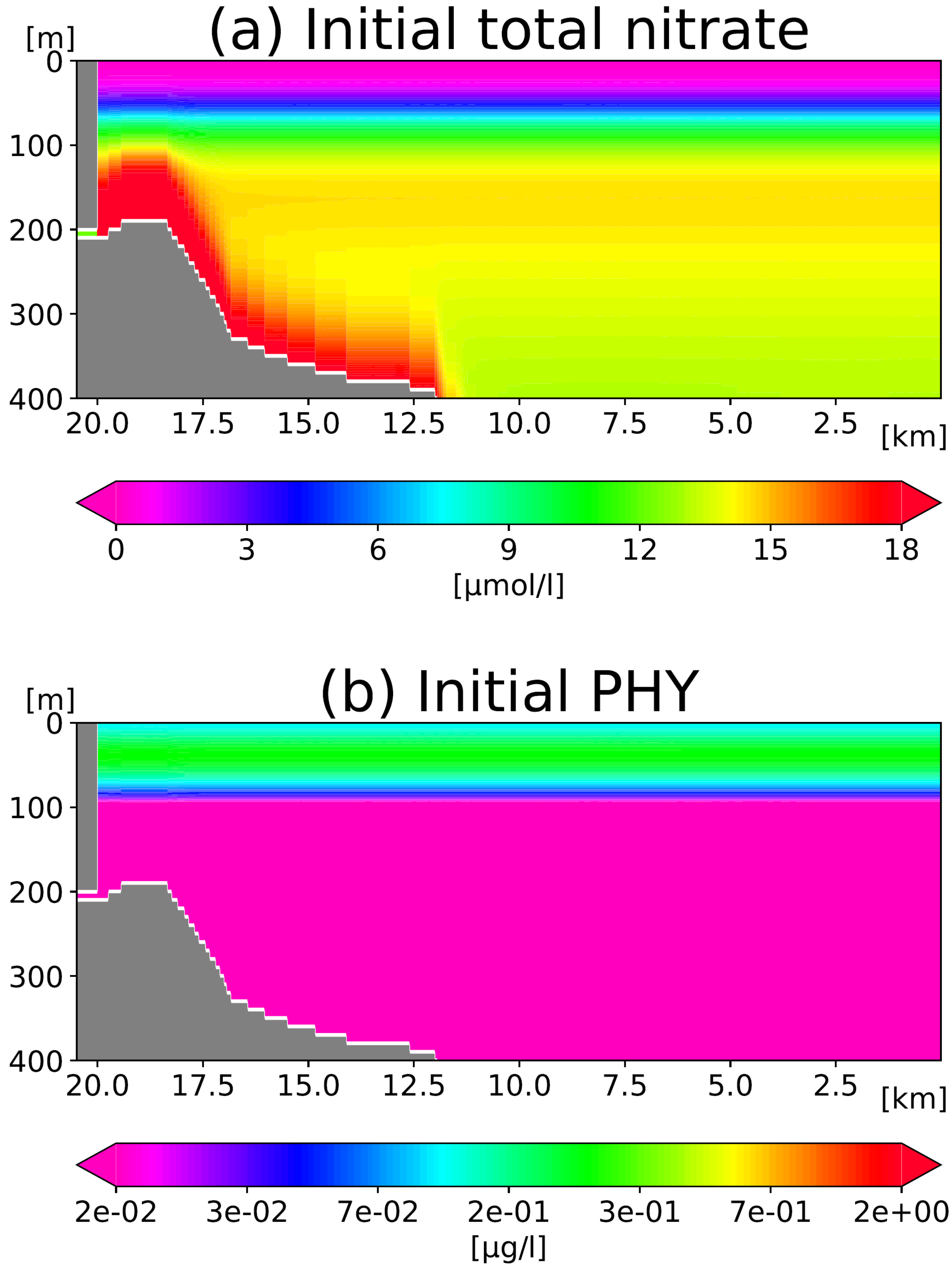


**Supplementary Figure 2.** (**a**) Initial condition (prior to meltwater discharge) of the total nitrate vertical distribution along a north–south section at the outlet. (**b**) Same as (a), but for phytoplankton.

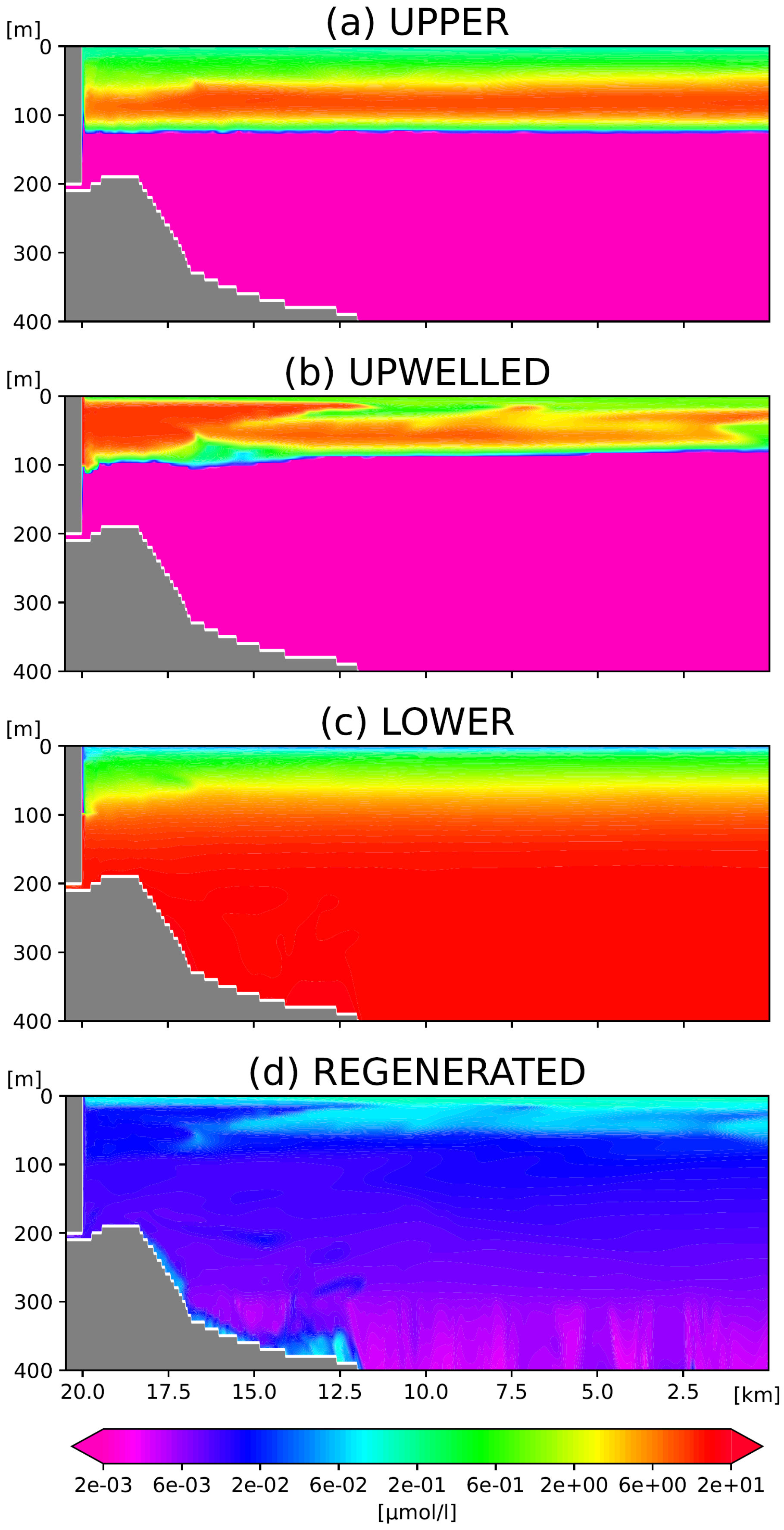


**Supplementary Figure 3.** Same as Figure 2, but for nutrients categorized by their source; (**a**) UPPER, (**b**) UPWELLED, (**c**) LOWER, and (**d**) REGENERATED nitrates.

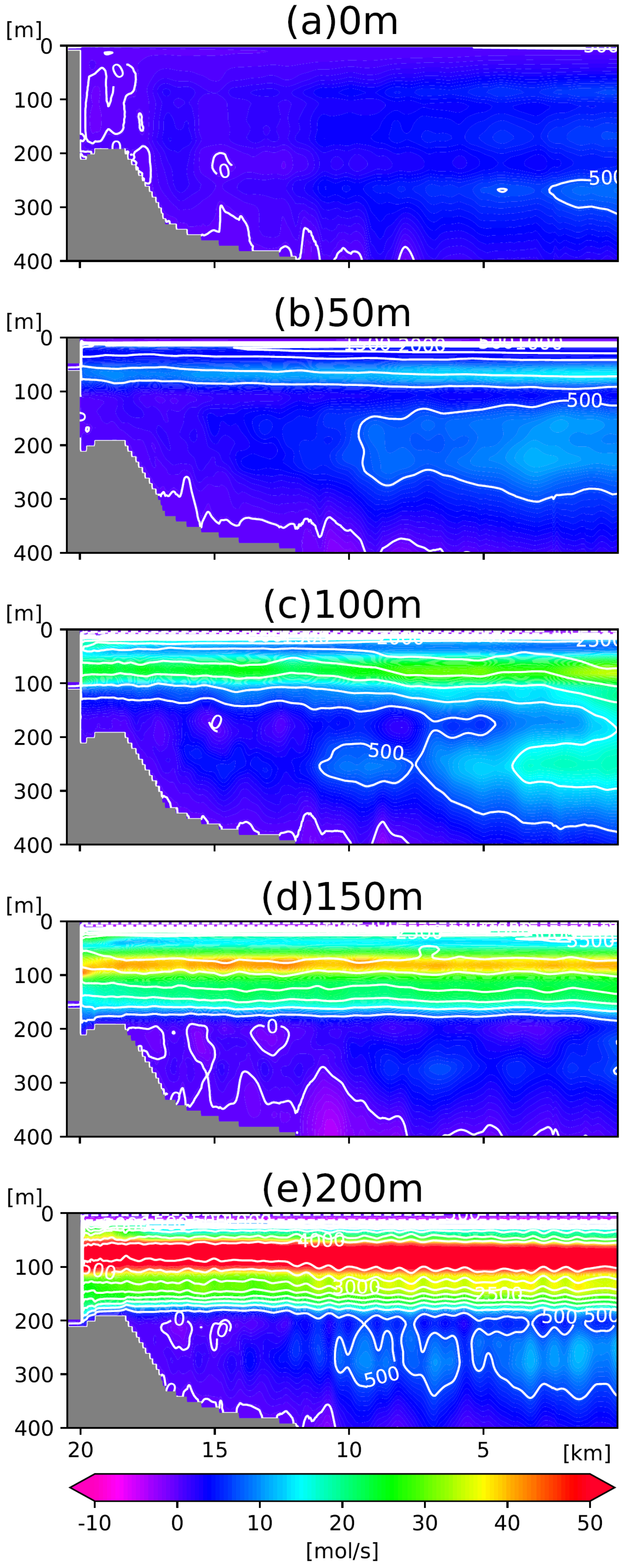


**Supplementary Figure 4.** Same as Figure 5 (a), but for outlet depths of (**a**) 0 m, (**b**) 50 m, (**c**) 100 m, (**d**) 150 m, and (**e**) 200 m and assuming a discharge rate of 100 $m^3/s$.

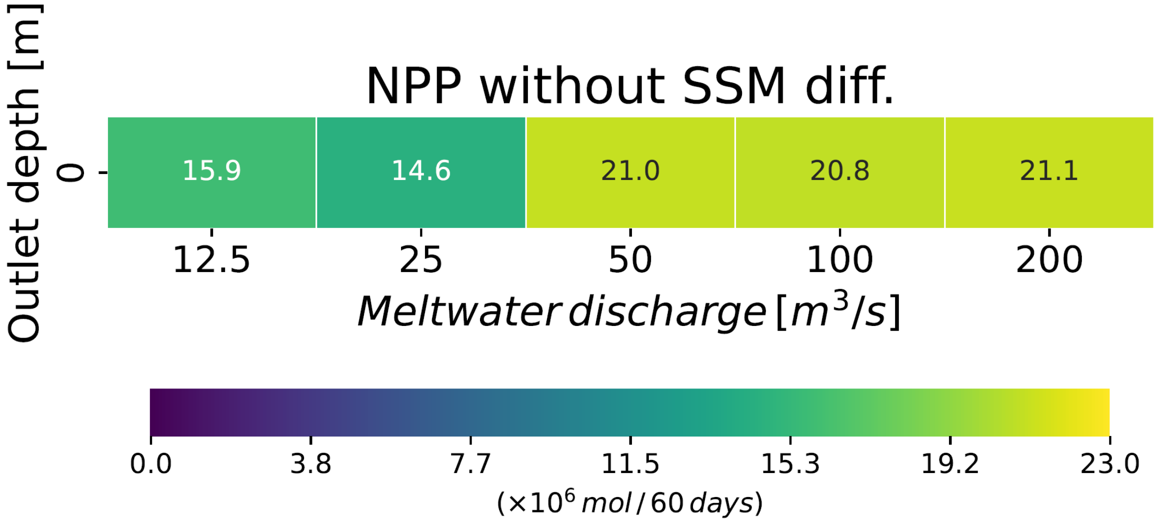


**Supplementary Figure 5.** Same as Figure 4 (a), but for cases without light attenuation by suspended sediment matter in sensitivity experiments, with respect to changes in meltwater discharge, and with a fixed fjord depth of 0 m.

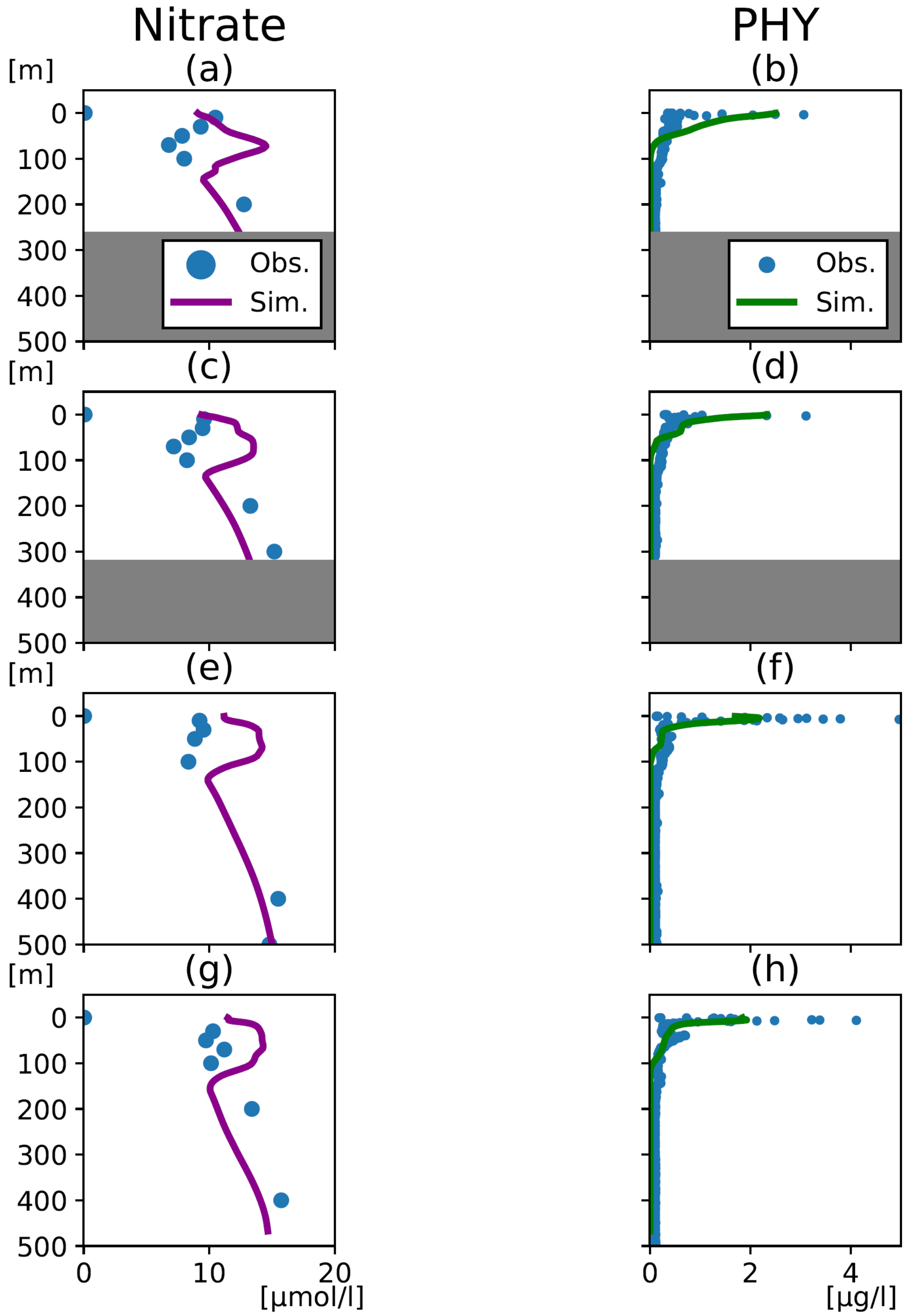


**Supplementary Figure 6.** (**a**) Vertical nitrate distributions of STD simulations (line) and observations at Bowdoin Fjord in July 2016 (dot). From top to bottom panels, dots show the results obtained at sites D2, D3, D4, and D5, as indicated in Kanna et al. (2018)[16]. The lower panels show the observed and simulated comparison points moving offshore. The results of the simulation were taken 7–8 days after the meltwater discharge had subsided for consistency with the timing of the field observation. Gray hatching represents the seafloor. (**b**) Same as (a), but for phytoplankton distributions. The observed phytoplankton is derived from in situ fluorescent measurement.

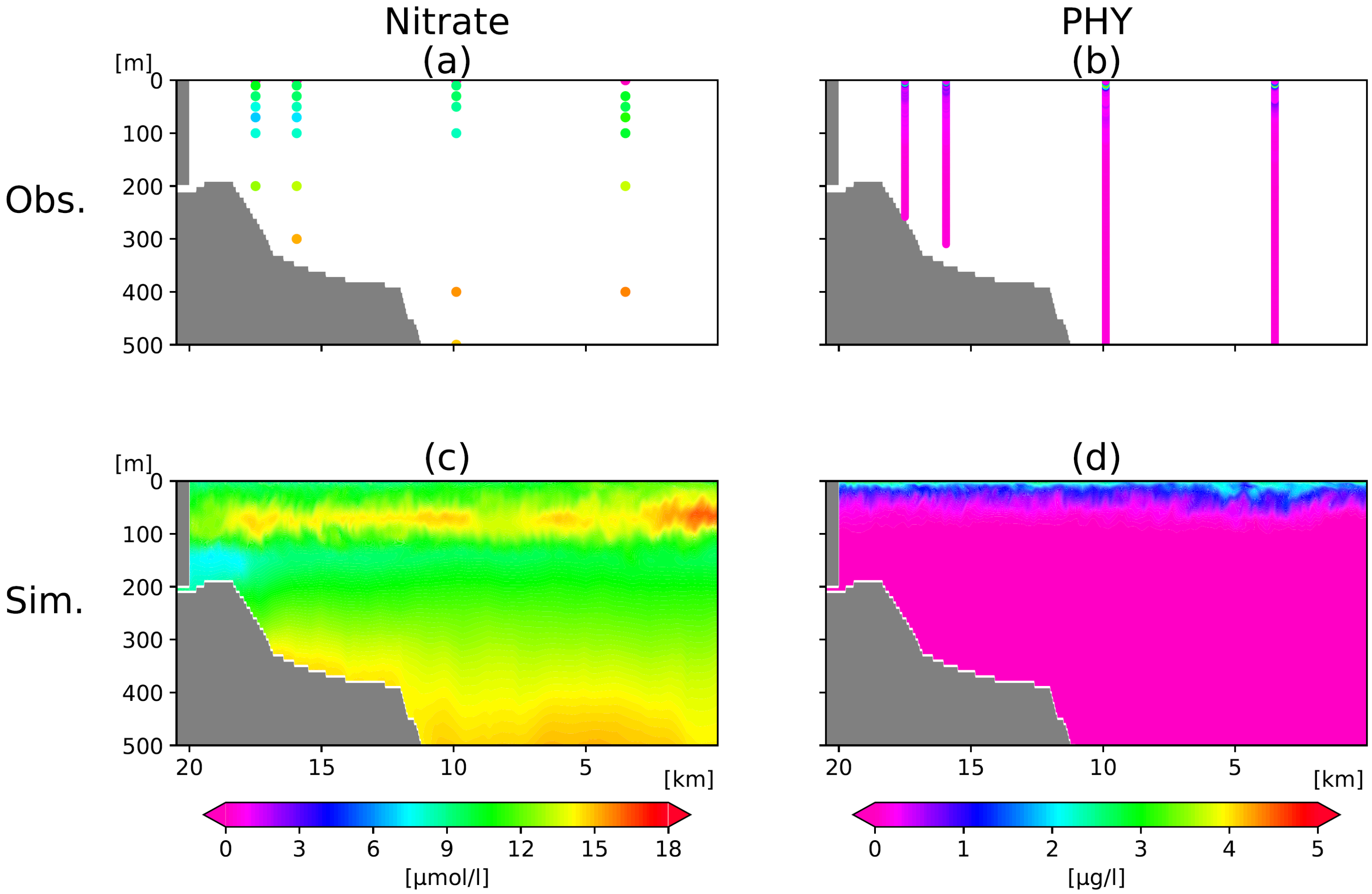


**Supplementary Figure 7.** Vertical cross-section of Bowdoin Fjord, showing the distributions of (**a**) observed nitrate, (**b**) observed phytoplankton, (**c**) simulated nitrate, and (**d**) simulated phytoplankton concentrations of STD. Locations of the observation and the corresponding time in the simulation are the same as those in Supplementary Figure 6.
